# Comparing The Effects of *Glycyrrhiza glabra* Root Extract, A Cyclooxygenase-2 Inhibitor (Celecoxib) and A Gonadotropin-Releasing Hormone Analog (Diphereline) In A Rat Model of Endometriosis

**DOI:** 10.22074/ijfs.2019.5446.

**Published:** 2019-01-06

**Authors:** Bahia Namavar Jahromi, Farnaz Farrokhnia, Nader Tanideh, Perikala Vijayananda Kumar, Mohammad Ebrahim Parsanezhad, Sanaz Alaee

**Affiliations:** 1 Infertility Research Center, Shiraz University of Medical Sciences, Shiraz, Iran; 2 Department of Obstetrics and Gynecology, School of Medicine, Shiraz University of Medical Sciences, Shiraz, Iran; 3 Student Research Center, Shiraz University of Medical Sciences, Shiraz, Iran; 4 Stem Cell Technology Research Center, Shiraz University of Medical Sciences, Shiraz, Iran; 5 Department of Pharmacology, School of Medicine, Shiraz University of Medical Sciences, Shiraz, Iran; 6 Department of Pathology, School of Medicine, Shiraz University of Medical Sciences, Shiraz, Iran; 7 Department of Reproductive Biology, School of Advanced Medical Sciences and Technologies, Shiraz University of Medical Sciences, Shiraz, Iran

**Keywords:** Celecoxib, Cyclooxygenase-2 Inhibitor, Endometriosis, Glycyrrhiza glabra, Gonadotropin Releasing Hormone

## Abstract

**Background:**

The purpose of this study was to compare the effects of *Glycyrrhiza glabra* (Licorice), a cyclooxyge- nase-2 inhibitor (Celecoxib) and a gonadotropin-releasing hormone analog (Diphereline®), with a control group on endometrial implants in rats.

**Materials and Methods:**

In this experimental study, endometriosis was induced in rats by auto transplantation and after confirmation, the rats were divided into 4 groups that were treated for 6 weeks with normal saline (0.5 ml/day, orally), licorice extract (3000 mg/kg/day, orally), celecoxib (50 mg/kg, twice a day, orally) or diphereline (3 mg/kg, intramuscularly). At the end of treatments, the mean area, volume, histopathology and hemosiderin-laden macrophage (HLM) counts of the endometrial implants were evaluated and compared among the four groups.

**Results:**

The mean area, volume and HLM counts of the implants in the licorice group were significantly lower than those of the control group (P<0.001). The histopathologic grades of endometrial implants were significantly decreased by licorice compared to the control group (P<0.001). There was no significant change in the mentioned parameters in rats treated with celecoxib compared to the control group. Diphereline was the most potent agent for suppressing the growth of endometrial implants in terms of all of the above-mentioned parameters.

**Conclusion:**

Licorice decreased the growth and histopathologic grades of auto-transplanted endometrial implants. However, while celcoxib had no significant effect, diphereline showed the highest potency for decreasing the endome- trial growth. Licorice may have the potential to be used as an alternative medication for the treatment of endometriosis.

## Introduction

Endometriosis, an estrogen-dependent inflammatory disease affecting 10-25% of women, is associated with significant reductions in fertility and is one of the most common benign gynecological diseases. Retrograde menstruation with subsequent adhesion formation, invasion, and neovascularization are believed to give rise to the occurrence of endometriosis lesions. The most common locations for endometrial implants are the ovaries, fossa ovarica, uterosacral ligaments, and posterior cul-de-sac (1). 

Although different medications are used to control endometriosis, their adverse effects following long-term use and recurrence of disease after discontinuation of therapy limit their applications. Additionally, these medications are not useful for endometriosis-associated infertility (2). Regarding the fact that no ideal medical treatment is available to control endometriosis, introducing new medical agents with minimal side effects and improved effectiveness for infertility treatment, are required.

Gonadotropin releasing hormone agonists (GnRHa) such as diphereline, as standard medications for the treatment of endometriosis, are able to induce inactivation and degeneration of endometrial implants via suppression of hypothalamic-pituitary-gonadal axis and ovarian estrogen production (3). GnRHa not only induces amenorrhea, but also may cause hot flush, depression, headache, hair loss, musculoskeletal stiffness, vaginal dryness and bone loss (4).

It is known that in women with endometriosis, the growth of endometrial cells within the peritoneal cavity is induced by inflammatory mechanisms (5); so, anti-inflammatory drugs are suggested to control endometriosis growth. Cyclooxygenase enzymes (COXs), known as prostaglandin-endoperoxide synthase, are responsible for formation of inflammatory mediators such as prostaglandins. COX-1 is expressed in almost all cells for maintenance of cell. COX-2 is produced at sites of inflammation, angiogenesis, and estrogenic cellular processes. Pharmacological inhibition of COX-2 was able to reduce the survival and growth of endometrial tissues at ectopic sites (6). NSAIDs (non-steroidal anti-inflammatory drugs) such as celecoxib, inhibit cyclooxygenase isoforms and induce gastrointestinal side effects (7). 

Using herbal medicine has always played a significant role in Iranian culture and civilization and some of these herbs have been recommended for treatment of infertility- related diseases. Licorice (*Glycyrrhiza glabra*), is one of the most widely used herbal drugs in Iranian traditional medicine. Licorice root contains triterpene, saponins, flavonoids, isoflavonoids, hydroxycoumarins, steroids and volatile oil. Licoricidin, is a potent compound isolated from licorice root (8). Studies showed that licoricidin is a selective COX-2 inhibitor and inhibits phospholipase A2 activity that is a critical enzyme involved in numerous inflammatory processes (9, 10). Licorice root with its anti- inflammatory/anti-platelet, antiviral, antifungal and mineralocorticoid functions has been used for the treatment of gastric ulcers, cough and bronchitis since the ancient times. Licorice is not recommended to be used for more than 6 weeks. Complications such as hypokalemia, hypernatremia, edema, hypertension and cardiac complaints are associated with long-term time use of licorice (8).

We hypothesized that licorice or celecoxib might be good candidates for treatment of endometriosis as an inflammatory condition. In the present study, we compared the effects of licorice, celecoxib and diphereline on the growth of endometrial implants in rats.

## Materials and Methods

In this experimental study, 48 mature female Sprague- Dawley rats (almost 8 weeks old, weighting 220 ± 20 g) were purchased from the Center of Comparative and Experimental Medicine at Shiraz University of Medical Sciences (SUMS), Shiraz, Iran. The animals were kept on 12 hours light: 12 hours dark cycles at a controlled temperature with free access to water and food. The animal experiments were performed according to the principles of the care and use of laboratory animals established by the National Institutes of Health, Bethesda, MD, USA, and approved by the Institutional Animal Ethics Committee at SUMS (No. 92-01-01-6869). These animal experiments were performed in the animal house of Shiraz University of Medical Sciences. To select the rats with normal estrous cycle, daily vaginal smears were taken and evaluated by a light microscope. Rats with three normal estrous cycles were used in the next steps.

### Preparation of licorice extract

Licorice roots were purchased from herbal stores in Shiraz, Iran). *Glycyrrhiza glabra* was preserved in herbarium after authentication by a botanist (Voucher No. PM 684). L. Licorice roots were air-dried, powdered and an alcoholic extract was produced by using ethanol (80%) and percolation method. Then, solvent was completely removed by drying under reduced pressure in a rotary evaporator. The extract was stored at 4°C until use.

### Induction of endometriosis

**Fig.1:**
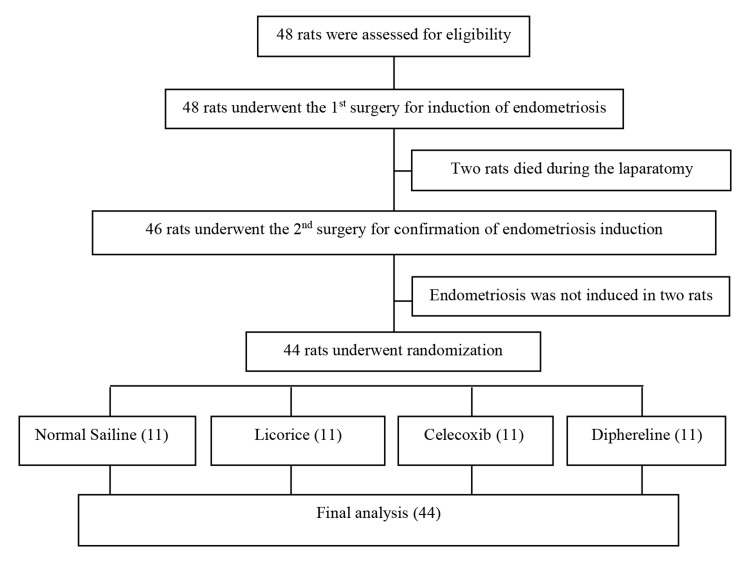
The flow diagram of the study.

Endometriosis was induced surgically using the method described by Vernon and Wilson with little modifications (11) (Fig .1). It should be mentioned that as the growth of endometriosis is estrogen-dependent, if induction of endometriosis is performed in an ovariectomized animal, estrogen supplementation is mandatory (11, 12). However similar to the previous researches, since in our study adult intact rats were used, we did not use exogenous estrogen for induction of endometriosis (11-13).

Briefly, all the female rats were anesthetized using ketamine hydrochloride 10% (100 mg/kg, Alfasan, Netherlands) and xylazine 2% (10 mg/kg, Alfasan, Netherlands). Then, rats’ abdomen was opened through a 4 cm midline incision starting from 1 cm below the xiphoid. The left horn of uterus was ligated at both the uterotubal and cervical junction ends and removed. A longitudinal cut was made through the uterine horn. By a punch biopsy, 4 round pieces of the distal part of uterine tissue were excised (4×4×1 mm) and placed in warm sterile saline 0.9%. Two implants were sutured with proline 5-0, one on the left and the other on the right side of the peritoneal cavity on the areas of well-visible vasculature with endometrial surface facing the peritoneum. Finally, the abdominal muscles, fascia and skin were sutured. Then, chlortetracycline (Cyclo Spray, Eurovet, UK) was sprayed on the incisions site and animals were allowed to recover from anesthesia. Two rats died due to hemorrhage at this stage. Six weeks after the first surgery, a second look laparotomy was performed and the viability of endometrial implants was confirmed by observation of good vascular supply and pinkish colored tissue in contrast to necrosis and fibrosis seen in two rejected cases as showed in Figure 1.

### Treatments

**Fig.2:**
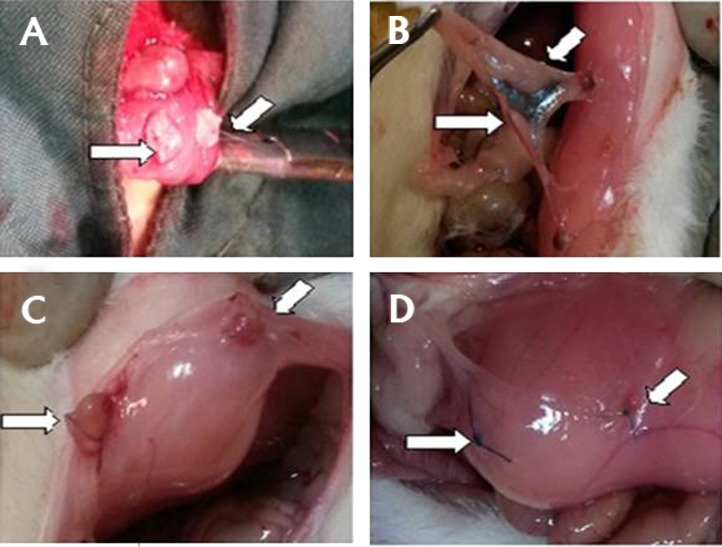
Endometrial implants in different times and groups. A. The first laparotomy: auto-transplant of endometrial implants on the peritoneum, B. The second look surgery: shows the adhesion bands and endometrial implants six weeks after induction of endometriosis, C. The third surgery: necropsy of a rat in the control group showing growth of implanted lesions of endometriosis, and D. The third surgery: necropsy of a rat in diphereline group showing regression of the implants.

At this stage, 44 female rats were divided into 4 groups (11 rats in each group). The control group was treated by 0.5 ml of saline 0.9%/day, the second group by licorice root extract (3000 mg/kg/day) and the third group took celecoxib (Damloran Razak Pharmaceutical Co., Iran, 50 mg/kg, twice a day, dissolved in 0.5 ml of saline 0.9%) for the next 6 weeks. All the treatments of these three groups were administered by oral gavage. The fourth group received a single IM injection of diphereline S.R. 11.25 mg (3 mg/kg, Ipsen, France). Six weeks after the treatments the rats were sacrificed and endometrial implants were evaluated as shown in Figure 2.

### Measuring area and volume of endometrial implants

The length, width and height of each implant were carefully measured using collis rulers by one researcher who was blinded to the treatment arms. The area of the endometrial implants in four groups was measured by multiplying length by width), and the volume was calculated by ellipsoid volume formulation (π/6×length×width×height). 

### Pathologic scoring of implants

All of the endometrial implants were fixed in formalin, placed in paraffin, cut into 5 µm sections, stained with hematoxylin-eosin and evaluated by the same pathologist. Photographs were taken by a digital camera (Sony, Japan). To classify the persistence of epithelial cells in grafts, the scoring system applied by Keenan et al. (14) was used with score 0 showing no epithelial layer, and scores 1, 2 and 3 show poorly, moderately and well-preserved epithelial layers, respectively. The percentage of hemosiderin-laden macrophages (HLMs) was also measured in all of the sections. The surgeon, pathologist, and the lab technicians were blinded to the groupings, medications, and specimens.

### Statistical analysis

For statistical analysis, the software SPSS 15 (SPSS Inc., Chicago, USA) was employed. To compare the mean area and the mean volume, ANOVA followed by Tukey HSD test was performed. To compare the histopathologic scoring, Kruskal-Wallis test and Mann-Whitney U test with Bonferroni correction were applied. A P<0.05 was considered significant.

**Table 1:** The mean area, volume and pathologic scores of implants in control, licorice, celecoxib and diphereline groups


Groups	Area (cm^2^)	Volume (cm^3^)	Pathologic score	Hemosiderin-laden macrophages

Control	42.94 ± 11.76	125.90 ± 11.69	2.5 ± 0.70	51.00 ± 9.90^c^
Licorice	27.57 ± 17.84^a^	90.86 ± 19.32^a^	1.90 ± 1.04	1.20 ± 1.07^d^
Celecoxib	39.87 ± 13.57	121.03 ± 7.08	2.44 ± 0.88	41.80 ± 6.4
Diphereline	8.60 ± 2.53^b^	11.00 ± 2.56^b^	0.54 ± 0.68^b^	1.2 ± 1.00


Data was shown as mean ± SD. P<0.05 were considered statistically significant. ^a^; Statistically significant differences between licorice and the control group, ^b^; Statistically significant differences between diphereline and the control group, ^c^; Statistically significant differences between control and other groups, and ^d^; Statistically significant differences between Licorice, diphereline and celecoxib group.

## Results

**Fig.3:**
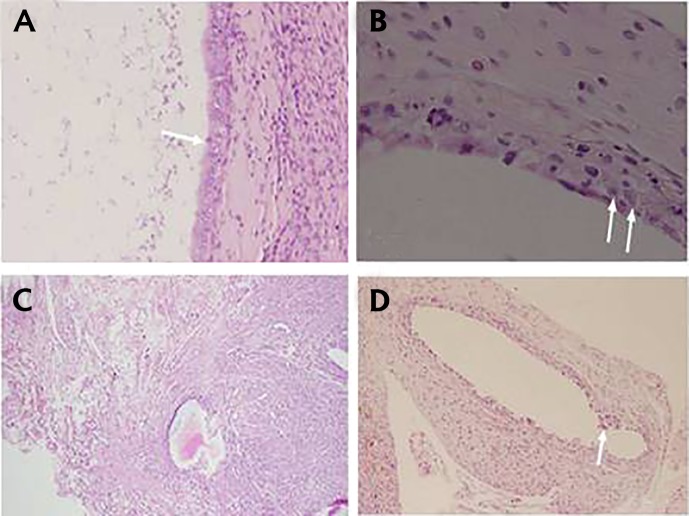
Specimens of the treated groups stained by hematoxylin-eosin. **A**. Well preserved epithelial layer of endometrial implants in the control group (grade 3) (scale bar: 50 µm), **B**. Poorly preserved epithelial layer of endometrial implants in the licorice group (grade 1) (scale bar: 50 µm), **C**. Moderately preserved epithelial layer of endometrial implants in licorice group (grade 2) (scale bar: 100 µm), and D. Poorly preserved epithelial layer of endometrial implants in diphereline group (grade 1) (scale bar: 100 µm). Arrows demonstrate epithelial layer of endometrial implants.

Two rats died during laparotomy due to hemorrhage and in two other rats the implants did not grow. The remaining 44 rats were divided into 4 groups and treated. In licorice group, the mean area and volume values of endometrial implants were significantly lower than those of the control group (P=0.042 and P<0.001, respectively) (Table 1). The mean area and volume of endometrial implants in the celecoxib group were lower compared to the control group, but the differences were not statistically significant (P= 0.953 and P=0.818, respectively). The mean area and volume of diphereline group were significantly lower compared to the control group (P<0.001 and P<0.001, respectively). The pathologic scores of the licorice and celecoxib groups were lower than those of the control group, but the differences were not statistically significant (P=0.221 and P=0.960, respectively). Poorly preserved epithelial layers were observed in diphereline group and the mean pathological score in this group, was significantly lower compared to the control group (P<0.001, Fig .3). 

**Fig.4:**
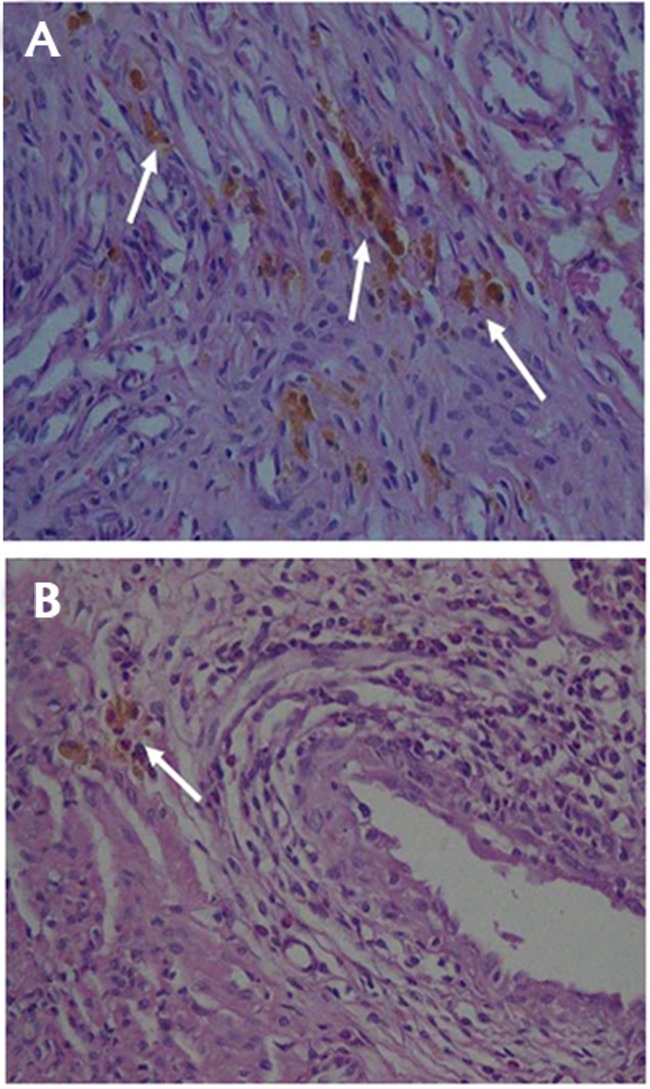
Hemosiderin-laden macrophages (HLMs) in the specimens of different groups. **A.** Control group and **B.** Licorice group (scale bar: 10 µm). Arrows indicate hemosiderin - laden macrophages.

The percentage of HLMs in endometrial implants of rats in celecoxib, licorice and diphereline group was significantly lower than that of the control group (P=0.004, P=0.000 and P=0.000, respectively). Also, the percentage of HLMs was significantly lower in licorice and diphereline group compared to celecoxib group (P<0.001 and P<0.001, respectively). The percentage of HLMs was not different between licorice and diphereline group (P=1.000, Fig .4).

## Discussion

We compared the effects of licorice, celecoxib, and diphereline in a rat model of endometriosis induced by auto-transplantation of endometrium on the peritoneal surface as a well-established method (11). Licorice decreased the growth of endometrial implants; celecoxib had no significant effect and diphereline had the highest potency in suppression of the endometrial growth. According to our knowledge, this is the first study on the effect of licorice on the endometrial implants.

Previous studies showed that glycyrrhetinic acid as a constituent of licorice extract, inhibits thrombin-induced platelet aggregation (9, 15) and has steroid-like anti-inflammatory effects similar to glucocorticoids (16, 17). Park and colleagues showed that administration of hexane/ ethanol extract of Glycyrrhiza uralensis to mice decreases cell proliferation, inhibits the expression of angiogenic and inflammatory proteins and induces cell cycle arrest or apoptosis (18). Also, they observed that licoricidin reduces macrophages number and tumor growth in the tumor microenvironment. In another study, it was shown that licoricidin inhibits the metastatic and invasive capacity of malignant prostate cancer cells in vitro (19). La et al. (20) reported that licoricidin suppresses the production of inflammatory cytokines. The anti-inflammatory property of licoricidin is due, in part, to the inhibition of phospholipase A2 activity, resulting in inhibition of cyclooxygenase activity and prostaglandin formation (9, 16, 17). Licoricidin also inhibits an isomer of platelet-activating factor and acetyltransferase resulting in an anti-inflammatory activity (21).

COX-2 overexpression has been detected in both eutopic and ectopic endometrium, and also in peritoneal macrophages derived from women with endometriosis (22, 23). In the family of selective COX-2 inhibitors, rofecoxib and valdecoxib, are no longer used because of their side effects but celecoxib with lower gastrointestinal problems is still used.

Histhopathologic slides prepared from endometriosis showed higher counts of HLMs that serve as an indirect evidence for diagnosis of endometriosis. As shown in our study, endometrial implants had normal growth with intact endometrial lining and more scattered foci of HLMs after treatment with celecoxib or normal saline. However, after taking licorice or diphereline, growth of endometrial implants were highly limited with lower HLMs. These findings are in favor of the potential therapeutic effect of licorice in suppression of endometrial implants growth.

In this study, celecoxib did not significantly reduce the growth of endometrial implants that was against our primary hypothesis. There are studies that showed that celecoxib, dexketoprofen trometamol or rofecoxib were able to cause regression and atrophy of endometriosis lesions (7, 21, 24). However, Hull et al. (25) showed that subcutaneous injection of nimesulide, a COX-2 inhibitor did not reduce the size and number of the endometriosis lesions that is in agreement with our results. We believe that ineffectiveness of celecoxib in our study might have been because of two reasons. First, it was previously shown that COX-2 immunostaining density was greater in ovarian endometrioma than in peritoneal implants and it was concluded that celecoxib might influence ovarian endometrioma more than peritoneum (26). Second, the celecoxib brand that we used was different from that of other studies.

Since endometriosis is a chronic disease and needs long-term treatment, complications of prolonged use of licorice such as hypokalemia, hypernatremia, edema, hypertension and cardiac complaints should be kept in mind before human application. It should be considered that the maximum permitted dosage of licorice root is 5 to 15gr/day and the duration of treatment should not exceed 6 weeks in humans (8). Studies on all components and fractions of licorice are also needed to discover its active component(s) and exact mechanism(s) of action to introduce a safe pharmacological agent with targeted effects and without adverse effects.

As a limitation of our study, we did not evaluate inflammatory markers such as white blood cells counts, nor interleukin-6 (IL-6), vascular endothelial growth factor (VEGF) and tumor necrosis factor-alpha (TNF-a) levels in the peripheral blood and peritoneal fluid before and after interventions to assess the anti-inflammatory properties of licorice.

We believe that licorice might have the potency to be used as a novel and excellent alternative in the management of endometriosis after in-depth investigations in animals and humans.

## Conclusion

Licorice decreased the growth and histopathologic grades of auto-transplanted endometrial implants. However, celecoxib had no significant effect and diphereline had the highest potency in reduction of the endometrial growth. 
